# Apoptosis of cardiomyocytes in diabetic cardiomyopathy involves overexpression of glycogen synthase kinase-3β

**DOI:** 10.1042/BSR20171307

**Published:** 2019-01-03

**Authors:** Wei Wu, Xingxing Liu, Longfei Han

**Affiliations:** Emergency Medicine, The First Hospital of China Medical University, Shenyang Liaoning Province, China

**Keywords:** apoptosis, diabetic cardiomyopathy, glycogen synthase kinase-3β, Phosphoinositide 3-kinases

## Abstract

To evaluate the role of glycogen synthase kinase-3β (GSK-3β) in the apoptosis of cardiomyocytes in diabetic cardiomyopathy (DCM). Diabetes mellitus (DM) in rats was induced by intraperitoneal injection of 1% streptozotocin (STZ), and lithium chloride (LiCl) was used to decrease the expression of GSK-3β. Hematoxylin/eosin (HE) staining and the terminal deoxyribonucleotide transferase-mediated dUTP-digoxigenin nick end labeling (TUNEL) assay was conducted to evaluate the pathological injury and apoptosis of cardiomyocytes respectively. Western blot was applied to detect the protein expressions of Cleaved-caspase 3, caspase 3, Bax and Bcl-2 in rat cardiomyocytes. Real-time polymerase chain reaction (RT-PCR) was applied to detect the gene expressions of phosphoinositide 3-kinases (PI3K), Akt, and GSK-3β in rat cardiomyocytes. DM-induced cardiomyocyte injuries, which were presented as capillary basement membrane thickening, interstitial fibrosis, cardiomyocyte hypertrophy and necrosis in HE staining and increased apoptosis detected by TUNEL assay. When comparing with the control group, the mRNA expression of PI3K and Akt in DM group obviously decreased but the mRNA expression of GSK-3β obviously elevated (*P* < 0.05). In addition, the ratio of Cleaved-caspase 3/caspase 3 and Bax/Bcl-2 were notably increased in DM group compared with control group (*P* < 0.05). LiCl, as an inhibitor of GSK-3 apparently reduced the expression of GSK-3β mRNA (*P* < 0.05) but not the PI3K and Akt comparing with the DM group. LiCl also attenuated the myocardial injury and apoptosis induced by DM. The myocardial injury induced by DM is associated with the up-regulation of GSK-3β. LiCl inhibited the expression of GSK-3β and myocardial apoptosis in diabetic myocardium.

## Introduction

Diabetes mellitus (DM) is one of the most important public health challenges and threat [1]. In 2015, the International Diabetes Federation (IDF) reported that there were 415 million adults with DM and the number will increase to 642 million by 2040 [2]. Along with the increasing prevalence of DM, cardiovascular complications secondary to DM have become the primary cause of death in DM. The current treatment of DM cannot reduce the incidence and progression of heart disease. So it is urgent to explore the potential mechanisms of cardiovascular complications in DM and find new targets to prevent its occurrence and development.

Diabetic cardiomyopathy (DCM), which was firstly proposed by Hamby [3], is one of the major heart complications in DM. DCM is a distinct cardiomyopathy other than myocardial injury induced by hypertension, coronary hand valvular heart disease. Epidemiological studies have found that more than 70% of DM patients in Western countries mainly died of cardiovascular disease, and the mortality was 2–3 times than non-DM population [4]. Early studies found that myocardial cell apoptosis and necrosis occurred in DCM and played a significant role in the development of DCM [5,6]. Glycogen synthase kinase-3β (GSK-3β) is closely related to apoptotic cells, and can induce apoptosis by activating apoptosis-related protease-caspase family (cysteine-containing aspartate-specific proteases) and Bax genes [7,8]. It has been shown that ischemia/reperfusion led to myocardial apoptosis through up-regulation GSK-3β gene expression, and apoptosis associated protein kinase activation of caspase 9, 3 [9]. High blood sugar induced apoptosis through activating phosphoinositide-3-kinase/protein kinase B (PI3K/Akt) signaling pathway [10,11] which was related to GSK-3β gene expression changes. The previous study has proved that lithium chloride (LiCl) conferred resistance to apoptosis in cancer cells through GSK-3β inhibition [12]. The present study aimed to evaluate the relationship between GSK-3β and myocardial apoptosis in DCM, and to provide a new direction for the prevention and treatment of DCM, based on a streptozotocin (STZ)-induced diabetic rat model and intervention of GSK-3β expression by LiCl.

## Materials and methods

### Reagents and kits

STZ (Sigma, St. Louis, MO), hydrogen peroxide (Solarbio Science & Technology CO., Ltd, Beijing, China), LiCl (Royalton, Dalian, Liaoning Province, China ), xylene, hematoxylin, eosin Y, alcohol, ethanol, terminal deoxyribonucleotide transferase-mediated dUTP-digoxigenin nick end labeling (TUNEL) assay kit (In Situ Cell Death Detection Kit, Roche, Mannheim, Germany), Triton X-100 (Beyotime Institute of Biotechnology, Nantong, Jiangsu Province, China), Total RNA Extraction Kit (Tiangen Biotech Co., Ltd., Beijing, China), Super M-MLV Reverse Transcriptase and 2×Power Taq PCR MasterMix (Bioteke Corporation, Beijing, China), cDNA synthesis kit (IScript, BioRad, U.S.A.), RNase solid scavengers (Tiandz, Inc., Beijing, China), Powder agarose (D1 LE, Hispanagar, Spain), 50×TAE buffer (50 mM EDTA, 2 M Tris base, and 1 M glacial acetic acid).

### Experimental study design

Four weeks old and healthy Wistar rats (*n* = 40) were selected and divided into three groups: control group (*n* = 10), DM group (*n* = 15), and DM+LiCl group (*n* = 15). After 1 week of adaptive feeding, rats in control group were given a standard diet, while rates in DM group and DM+LiCl group were given high-calorie diet for 4 weeks. Each 1 kg high-calorie diet included 0.51 kg standard feed, 0.20 kg lard, 0.28 kg sucrose, 0.01 kg cholesterol and 0.0025 kg bile salts. The comparison of a standard diet with high-calorie feed was shown in Table 1. Rats in DM group and DM+LiCl group received an intraperitoneal injection of 30 mg/kg 1% STZ after fasting for 24 h, while rats in control group were injected with equal volume of buffer as a control group (sodium citrate/citrate, pH 4.2). Both blood and urine glucose were detected after 24 and 48 h, and the detailed information of fasting blood glucose and glucose tolerance in each group were shown in Table 2. A successful diabetic model was defined as fasting blood glucose >11.1 mmol/l, postprandial blood glucose >16.7 mmol/l, urine sugar ≥ +++, and diabetic symptoms such as overeating, overdrinking, polyuria. Unsuccessful modeling rats received another injection of 15 mg/kg 1% STZ after fasting for 24 h and reevaluated according to the above criteria. After molding, the rats in DM+LiCl group received 15 mg/kg LiCl (8 mg/ml) intraperitoneally on the next day for 1 week, while the rats in control group and B were given the equal volume of normal saline. After 1 week, the rats were killed, and the one part of heart tissues were fixed in 10% formalin, and others were cut into pieces and stored in −80°C refrigerators for further use.

**Table 1:** The proportion of nutrient components in rat standard feed and high calorie feed

Ingredients	Standard (%)	High calorie (%)
Protein (%)	16	14
Fat (%)	8	25
Carbohydrates (%)	50	51
Trace elements and others (%)	22	10
Total calories (kJ)	14	20

**Table 2:** Fasting blood glucose (FBG) and glucose tolerance (mean±SD)

Groups	FBG at 24 h (mmol/l)	2 h postprandial blood glucose (mmol/l)	FBG at 48 h (mmol/l)
Control group	5.16 ± 0.33	5.45 ± 0.48	5.22 ± 0.38
DM group	15 ± 15.34*	28.63 ± 2.35*	15 ± 16.15*
DM+LiCl group	15 ± 16.61*	29.34 ± 2.18*	15 ± 16.65*

Notes: * *P* < 0.05 vs. control group

### Histopathological examination

Heart tissues were incubated in 10% formalin for 24–72 h and then embedded in paraffin. After sectioned (5 µm), the samples were stained with hematoxylin and eosin (HE) staining. At last, the slides were examined using the Olympus IX70 light microscope (Olympus Optical Co. Ltd., Japan) by two experienced pathologists who were blinded to the treatment groups. The myocardial damages were graded using a scoring system reported by Lobo Filho [13]. This evaluating system provided myocardial injury scores from 0 to 3: no change (grade 0); focal myocyte damage or small multifocal degeneration with slight degree of inflammation (grade 1, mild); extensive myofibrillar degeneration and diffuse inflammatory process (grade 2, moderate ); necrosis with diffuse inflammatory process (grade 3, severe).

### TUNEL assay

Briefly, The samples were fixed in 50 μl 0.1% Triton X-100 at room temperature for 8 min and then closed by adding 50 μl 3% H_2_O_2_ for 10 min. Rinsed with PBS, 3 × 5 min after each incubation. Then, the slides were incubated in 50 μl TUNEL reaction mixture and placed in a humidified box and in the dark for 60 min at 37°C and then washed with PBS three times for 5 min. To detect the nuclei, the samples were incubated with DAPI for 2 min at room temperature in the dark and then washed with PBS three times. At last the slides were observed with an Olympus BX51 Fluorescence microscope (Olympus Optical Co. Ltd., Japan). The severity of apoptosis was evaluated by the percentage of the TUNEL-positive cells from four randomly selected fields.

### Western blot analysis

The total protein was extracted using a protein extraction assay kit (KeyGen, Jiangsu, China) according to the manufacturer’s instructions. Total protein centrifuged (Cence, Changsha, China) at 12000 rpm for 20 min at 4°C and the supernatants were used for analyzing. The protein extract concentration was determined using a BCA protein assay kit (Solarbio, Beijing, China) in accordance with the manufacturer’s approach. Then equal amounts of protein extracts were subjected to electrophorese using 8–12% sodium dodecyl sulfate–polyacrylamide gel electrophoresis (SDS-PAGE) and transferred to PVDF membranes (Millipore, MA, U.S.A.). Subsequently, the membranes were blocked in 5% nonfat milk at room temperature for 1 h and incubated with the respective primary antibody overnight at 4°C. Then the membranes were incubated at room temperature with anti-rabbit secondary antibody conjugated to horseradish peroxidase (diluted 1:8000) for 2 h. The blotted membrane was detected by enhanced chemiluminescence method with ECL Western Blotting Detection Substrate (Biotool, Houston, U.S.A.). Tannon Image 1.0 software were used to densitometry analysis. β-actin was used as an internal control. Primary antibodies against caspase 3 (diluted 1:1000), Cleaved-caspase 3 (diluted 1:500), Bcl-2 (diluted 1:1000) and Bax (diluted 1:1000) were obtained from Sangon Biotech Co., Ltd., Shanghai, China. Primary antibody against β-actin (diluted 1:1000) were purchased from Zhongshang Goldenbridge Bio, Beijing, China.

### Real-time polymerase chain reaction (RT-PCR)

The total RNA extraction kit (Tiangen Biotech Co., Ltd., Beijing, China) was used to isolate total RNA. The cDNA synthesis kit (IScript, BioRad, U.S.A.) was used to synthesize complementary DNA from a 1.5 μg total RNA. We carried out the Real-time in the MX3000p PCR system (Stratagene, Europe) using 2×Power Taq PCR MasterMix (Bioteke Corporation, Beijing, China). The detailed information about primers used for real-time polymerase chain reaction (RT-PCR) was showed in Table 3. Through comparing the threshold cycle ratios between the candidate genes and β-actin, the data were normalized and then analyzed by the comparative CT method [14].

**Table 3:** Oligonucleotide primer sets for RT-PCR

Name	Sequence (5′–3′)	Length	Tm	Size (bp)
PI3K F	GATTTCGTATCCACCTGTCC	20	54.1	222
PI3K R	GCCTCTAATCTTCTCCCTCT	20	52.0	
AktF	GCTCTTCTTCCACCTGTCTCG	21	58.9	184
Akt R	CAGCCCGAAGTCCGTTATCT	20	59.3	
GSK-3β F	CACCTGCCCTCTTCAACTT	19	54.7	161
GSK-3β R	ATTGGTCTGTCCACGGTCT	19	54.3	
β-actin F	GGAGATTACTGCCCTGGCTCCTAGC	25	60.1	155
β-actin R	GGCCGGACTCATCGTACTCCTGCTT	25	62	

### Statistical analysis

Statistical Package for Student (SPSS) software version 24.0 (SPSS Inc., Chicago, IL, U.S.A.) was used for all statistical analysis. Continuous variables were expressed as the mean ± standard deviation (SD). We compared data between the three groups by Kruskal–Wallis test accompanied with Dunns’ multiple comparisons (if nonparametric tests) or one-way analysis of variance (ANOVA) followed by Student–Newman–Keul’s (SNK) test (if parametric tests). *P* < 0.05 was considered to be statistically significant.

### Ethical approval statement

The present study was approved by the ethical committee of The first hospital of China medical university.

## Results

### LiCl attenuated myocardial injury induced by DM

**Figure 1:**
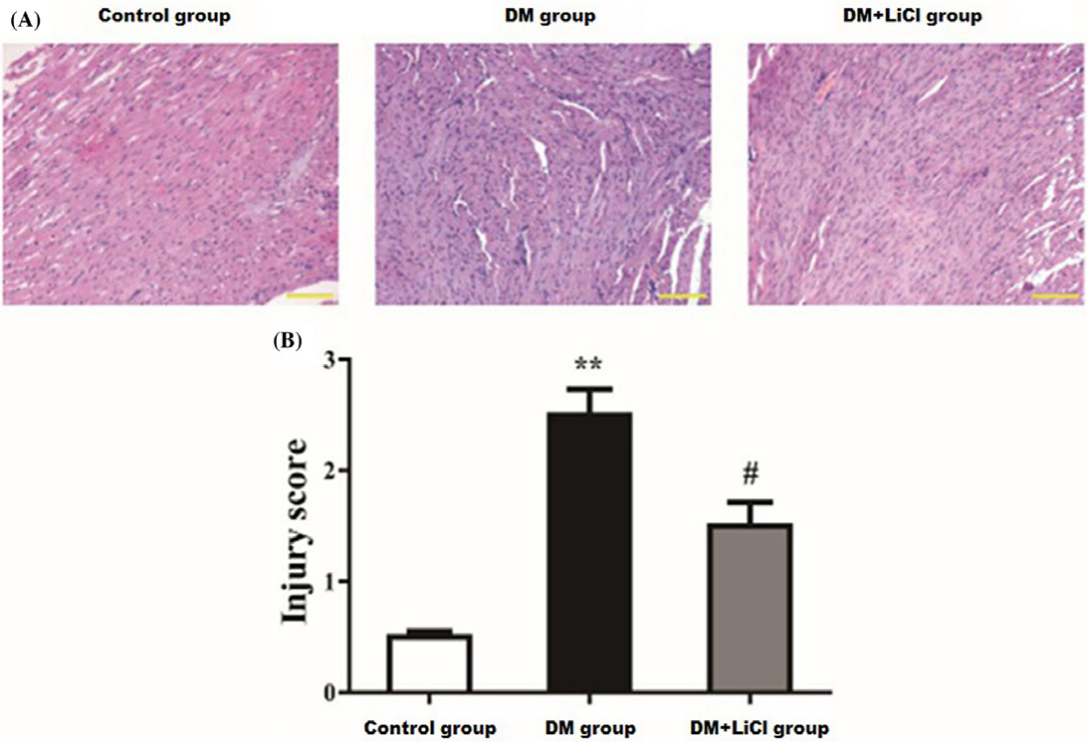
Histopathological change of the myocardium and the injury scores (**A**) HE staining of the myocardium; (**B**) myocardial injury score. Scale bar represents 100 μm (original magnification, 200×); ** *P* < 0.01 vs. control group; ^#^*P* < 0.05 vs. DM group.

The myocardial cells in control group arranged in neat with uniform size and shapes (Figure 1A). But in DM group, there were disordered cardiomyocytes, irregular nuclear size, capillary basement membrane thickening, interstitial fibrosis, cardiomyocyte hypertrophy and necrosis (Figure 1A). LiCl attenuated the above myocardial injury in DM+LiCl group (Figure 1A). The myocardial injury score was higher in DM group than that in control group (*P* < 0.01), and LiCl significantly decreased the injury score in DM+LiCl group than DM group (*P* < 0.05) (Figure 1B).

### The effect of LiCl on the levels of fasting blood glucose and glucose tolerance

As shown in Table 2, the levels of FBG at 24 h in rats of DM group were obviously increased compared with that in control rats (15±15.34 vs. 5.16±0.33, *P* < 0.05). However, there were no significant difference in FBG levels at 24 h between DM and DM+LiCl group (15±16.61 vs. 15±15.34). Similarly, the levels of FBG at 48 h were significantly increased in the DM group compared with the normal group (15±16.15 vs. 5.22±0.38, *P* < 0.05). However, there were no significant difference in FBG levels at 24 h between DM and DM+LiCl group (15±16.65 vs. 15±16.15). In addition, we also measured 2 h postprandial blood glucose levels in rats of each group. As shown in Table 2, the levels of 2 h postprandial blood glucose in DM group were notably higher than those of control group (28.63±2.35 vs. 5.45±0.48, *P* < 0.05), which were no obvious difference after LiCl treatment (29.34±2.18 vs. 28.63±2.35).

### LiCl attenuated myocardial apoptosis induced by DM

**Figure 2:**
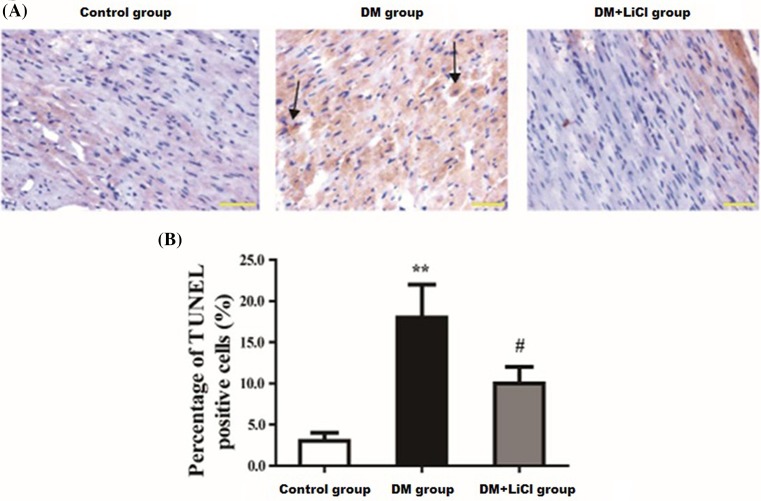
Comparison of myocardial apoptosis induced by diabetes (**A**) Representative photomicrographs of TUNEL staining. (**B**) Percentages of apoptotic cells of the myocardium. Scale bar represents 50 μm (original magnification, 400×) (arrows pointed apoptotic cells). **P < 0.01 vs. control group; ^#^*P* < 0.05 vs. DM group.

As shown in Figure 2A, in TUNEL staining, the nucleus of positive apoptotic cells was purple-black. The cells in the control group were in good condition, the cells were round, the shape of the cytoplasm was regular, and only a few distinct purple-black cells were seen in the nucleus. Compared with control group, many of the nucleus in the model group were deeply stained purple-black. The cell gap was enlarged. The apoptotic cells become smaller and deformed. The volume of necrotic cells swelled, and the cell membrane was damaged or lysed into fragments. Exfoliated cell debris was observed in the DM+LiCI group, but the cell morphology was significantly better than the model group. As were shown in Figure 2B, the percentage of TUNEL positive cells in DM group was significantly increased than control group (16.67% ± 3.68% vs. 3.31% ± 1.27%, respectively, *P* < 0.01). LiCl significantly decreased the rate of apoptotic cells in DM+LiCl group than DM group (9.59 ± 2.31 vs.16.67 ± 3.68, respectively, *P* < 0.05).

### The protein expression of Bax, Bcl-2, Cleaved-caspase 3 and caspase 3

**Figure 3:**
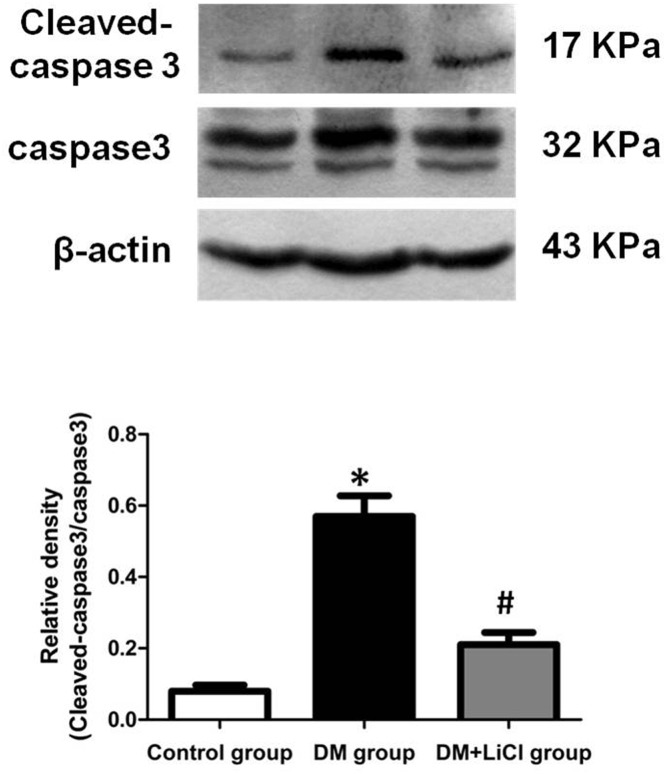
Comparison of expression levels of Cleaved-caspase 3 and caspase 3 protein *P < 0.05 vs. control group; ^#^*P* < 0.05 vs. DM group.

**Figure 4:**
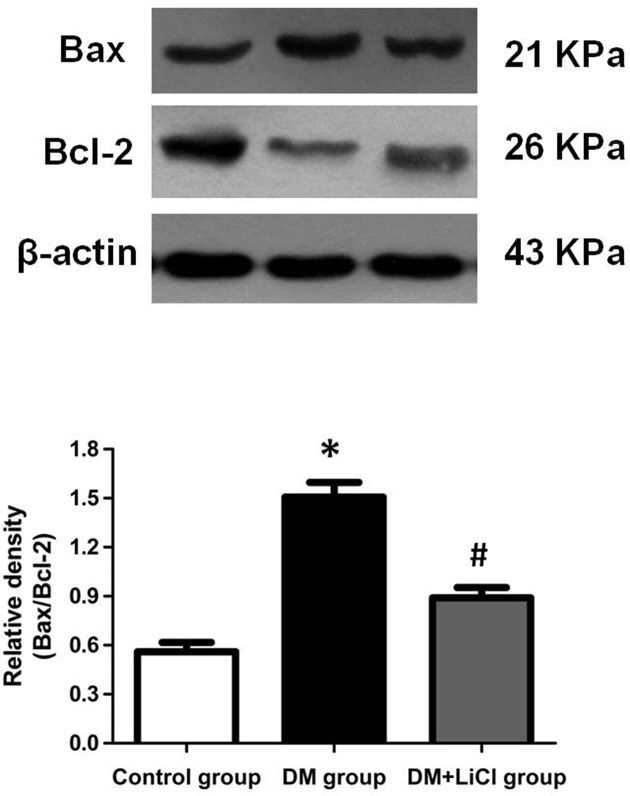
Comparison of expression levels of Bax and Bcl-2 protein *P  < 0.05 vs. control group; ^#^*P *< 0.05 vs. DM group.

To investigate the effects of LiCl on apoptosis in DM rats, the expression levels of Cleaved-caspase 3, caspase 3, Bax, and Bcl-2 protein were determined by Western blot. As shown in Figure 3, the ratio of Cleaved-caspase 3/caspase 3 protein expression levels of DM rats was significantly increased when compared with the control group (*P* < 0.05), which was markedly decreased after LiCl treatment (*P* < 0.05). In addition, compared with control group, the ratio of Bax/Bcl-2 protein expression levels of DM rats was significantly increased (*P* < 0.05), which was obviously inhibited by treatment with LiCl (*P* < 0.05) (Figure 4).

### The mRNA expression of PI3K, Akt and GSK-3β

**Figure 5:**
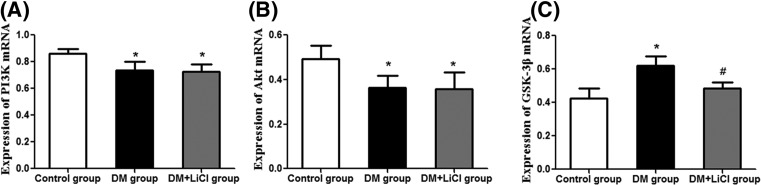
Comparison of expression levels of PI3K, Akt and GSK-3β mRNA *P < 0.05 vs. control group; ^#^*P* < 0.05 vs. DM group.

There were significantly lower expression levels of PI3K and Akt mRNA in DM group and DM+LiCl group than control group (*P *< 0.05) (Figure 5A,B). But the mRNA expression levels of GSK-3β in DM group and DM+LiCl group were significantly increased than control group (*P* < 0.05) (Figure 5C). LiCl significantly reduced the mRNA expression level of GSK-3β in DM+LiCl group than DM group (*P* < 0.05) (Figure 5C).

## Discussion

GSK-3β plays a significant role in cell growth, differentiation, apoptosis, signal transduction and other life activities [15]. More and more studies showed that many factors could activate GSK-3β and induce apoptosis [16–21]. In a variety of tissue cells and tumor cells, the activation of GSK-3β may further activate apoptosis-related protein kinase family caspase 3 and caspase 9, induce decomposition of nucleoprotein and shear fracture of DNA strand, and ultimately mediate apoptosis [22]. A previous study proved that the activity of GSK-3β in diabetes was as twice as that of non-diabetic patients [23]. In the present experiment, results showed that the gene expression of GSK-3β in DM group and DM+LiCl group significantly increased compared with control group, and the apoptotic rate also significantly increased. Furthermore, the ratio of Cleaved-caspase 3/caspase 3 and Bax/Bcl-2 protein in DM group and DM+LiCl group were also significantly increased when compared with control group. It can be inferred that the myocardial apoptosis in diabetes may involve the up-regulation expression of GSK-3β and the increased ratio of Cleaved-caspase 3/caspase 3 and Bax/Bcl-2 in myocardial cells. The expression of GSK-3β was up-regulated in high glucose-induced cardiomyocyte apoptosis in neonatal rats, while GSK-3β gene expression and cardiomyocyte apoptosis were significantly decreased under LiCl intervention, which indicating that the apoptosis of cardiomyocytes is related to the up-regulation of GSK-3β gene expression [24]. LiCl increases phosphorylate of GSK-3β at the site of serine 9, and thereby inhibits GSK-3β activity. In order to further clarify the above inference, the present study investigated the effect of GSK-3β gene expression and cardiomyocyte apoptosis by LiCl intervention. The results showed that the expression of GSK-3β in DM+LiCl group was significantly lower than that in DM group, and the apoptosis of cardiomyocytes was also significantly decreased, further confirming the up-regulation of cardiomyocyte apoptosis and GSK-3β gene expression in diabetic cardiomyocytes.

Phosphoinositide-3-kinase/protein kinase B (PI3K/Akt) signal transduction pathway is one of the critical pathways of cell membrane receptor signaling to intracellular conduction [25] and plays an anti-apoptotic effect in the heart, kidney, liver, brain and other organs [26]. GSK-3β is a downstream signaling molecule of Akt. The activated PI3K/Akt signal transduction pathway inhibits the activity of GSK-3β by phosphorylating the serine 9 residue of GSK-3β [27,21] and exerts an anti-apoptotic effect [28]. It has been demonstrated that Erythropoietin reduces myocardial ischemia-reperfusion injury, and the activation of PI3K/Akt signaling pathway and down-regulation of GSK-3β gene expression were required [29]. When the activation of PI3K/Akt pathway was specifically inhibited, the gene expression of GSK-3β increased and the apoptosis significantly increased [30]. After detecting the expression of PI3K, Akt and GSK-3β by RT-PCR, we found that the expression of PI3K and Akt in DM group and DM+LiCl group was significantly lower than that in control group, but the expression of GSK-3β was significantly increased. This suggests that elevated gene expression of GSK-3β is associated with low expression of PI3K / Akt signaling protein, and high glucose inhibits the activation of PI3K/Akt signaling pathway in cardiomyopathy, reduced the phosphorylation of GSK-3β, up-regulated the expression of GSK-3β and ultimately induced cardiomyocyte apoptosis.

In conclusion, the pathologic change caused by DM may lose the inhibition of GSK-3β through PI3K/Akt signal pathway, which induces diabetic myocardial apoptosis. LiCl inhibited the up-regulation of GSK-3β expression and myocardial apoptosis in diabetic myocardium.

## Abbreviations

DCM: diabetic cardiomyopathy
DM: diabetes mellitus
FBG: fasting blood glucose
GSK-3β: glycogen synthase kinase-3β
HE: hematoxylin/eosin
LiCl: lithium chloride
PI3K/Akt: phosphoinositide-3-kinase/protein kinase B
RT-PCR: real-time polymerase chain reaction
STZ: streptozotocin
TUNEL: transferase-mediated dUTP-digoxigenin nick end labeling
